# Chemical composition and antibacterial properties of essential oil and fatty acids of different parts of *Ligularia persica* Boiss

**Published:** 2016

**Authors:** Maryam Mohadjerani, Rahman Hosseinzadeh, Maryam Hosseini

**Affiliations:** 1 *Department of Molecular and Cell Biology, Faculty of basic Sciences, University of Mazandaran, Babolsar, Iran*; 2 *Department of Organic Chemistry, Faculty of Chemistry, University of Mazandaran, Babolsar, Iran*

**Keywords:** *Ligularia persica*, *Asteraceae*, *Essential oil composition*, *Fatty acids*, *Anti-bacterial*

## Abstract

**Objective:**

The objective of this research was to investigate the chemical composition and antibacterial activities of the fatty acids and essential oil from various parts of *Ligularia persica* Boiss (*L. persica*) growing wild in north of Iran.

**Materials and Methods:**

Essential oils were extracted by using Clevenger-type apparatus. Antibacterial activity was tested on two Gram-positive and two Gram-negative bacteria by using micro dilution method.

**Results:**

GC and GC∕MS analysis of the oils resulted in detection of 94%, 96%, 93%, 99% of the total essential oil of flowers, stems, roots and leaves, respectively. The main components of flowers oil were cis-ocimene (15.4%), β-myrcene (4.4%), β-ocimene (3.9%), and γ-terpinene (5.0%). The major constituents of stems oil were β-phellandrene (5.4%), β-cymene (7.0%), valencene (3.9%). The main compounds of root oil were fukinanolid (17.0%), α-phellandrene (11.5%) and Β-selinene (5.0%) and in the case of leaves oil were cis-ocimene (4.8%), β-ocimene (4.9%), and linolenic acid methyl ester (4.7%). An analysis by GC-FID and GC-MS on the fatty-acid composition of the different parts of *L. persica* showed that major components were linoleic acid (11.3-31.6%), linolenic acid (4.7-21.8%) and palmitic acid (7.2-23.2%). Saturated fatty acids were found in lower amounts than unsaturated ones. The least minimum inhibition concentration (MIC) of the *L. persica* was 7.16 μg/ml against *Pseudomonas aeruginosa*.

**Conclusion:**

Our study indicated that the essential oil from *L. persica* stems and flowers showed high inhibitory effect on the Gram negative bacteria. The results also showed that fatty acids from the stems and leaves contained a high amount of poly-unsaturated fatty acids (PUFAs).

## Introduction

For centuries, essential oils have been used for the treatment of infections and diseases, in different parts of the world (Rios and Recio, 2005). Nowadays, the use of essential oils is growing and there is a noticeable range application for them (e.g. in food and beverages industry, as fragrances in perfumes and cosmetics) but the oils also cover a broad spectrum of biological activities which has aroused the researchers’ interest. In the past two decades, there has been a lot of research to study the antimicrobial activity of essential oils. The main constituents of some plant essential oils are thymol, carvacrol, linalool and eugenol that have been shown to have a wide spectrum of antimicrobial activities (Kalemba and Kunicke, 2003; Dorman and Deans, 2000). Recently, the antibacterial properties and potential use of essential oils in foods have been investigated (Burt, 2004). 

Antimicrobial activities of spices and herbs have been known for several centuries (Bagamboula et al., 2003). Essential oils and their components are becoming increasingly popular as natural antimicrobial agents to be used for a wide variety of purposes, including food preservation, complementary medicine and natural therapeutics. At present, essential oils are used by the flavoring industry for flavor enhancement and for their antioxidant effects (Cosentino et al., 2003). Fatty acids have also a wide range of functions (Elias, 1983). For example, some polyunsaturated fatty acids such as nervonic acid, linoleic acid and arachidic acid are vital for human growth (Carvalho et al., 2006).

*Ligularia persica* Boiss (*L. persica*(. is an important species of Compositae family. According to Flora Iranica, there is only one species of *Ligularia* in Iran that is endemic of north of Iran. The local names of this genus are ^″^Zabantala^″^ and ^″^Pirsonbol^″^ (Rechinger, 1989). *Ligularia* species are used in traditional medicines such as treatment of coughs, inflammations, jaundice, scarlet fever, rheumatoid arthritis, and hepatic diseases (Xie et al., 2010). Up to now, several phytochemical studies have identified various compounds such as steroids, alkaloids, flavonoids, lignans, sesquiterpenoids, and terpenoids in *ligularia* species (Yang et al., 2011). 

The secondary metabolites reported from *L. persica* have anti-bacterial, anti-lung cancer, anti- stomach cancer, anti-hepatotoxicity, anti-thrombotic, anti-coagulation and anti-insect activity (Yang et al., 2011). Extraction of roots of *L. persica* and chromatographic separation revealed one new derivative of tovarol, four new derivatives of shiromodiol, α - and β - eudesmol, bakkenolide A and four known eremophilane derivatives (Marco et al., 1991). There is a report on chemical composition and antimicrobial activities of aerial parts of *L. persica* in the literature (Mirjalili and Yousefzadi, 2012). However, no previous work has been conducted on different part of this plant. Also, there is no report on the fatty acids composition and antibacterial activity of the different parts of *L. persica* essential oils. Therefore, the aim of this research is to analyze the chemical constituents and fatty acids of different parts of *L. persica* and antibacterial activity of the essential oils of different parts of *L. persica* was then investigated and discussed.

## Materials and methods

**Plant Material**

*L* *.* *persica *was collected during the flowering stage in July 2012 from Pole Zangule located in central Alborz Mountains (Mazandaran province, North of Iran). The specimen was identified and authenticated by a taxonomist, Dr Alireza Naqinezhad, and a voucher herbarium specimen was deposited in the herbarium of the Department of Biology, University of Mazandaran (No. 1505). The plant material was air-dried at room temperature and protected from light for one week.

**Isolation of essential oil**

Different parts of *L. persica *(50 g) were subjected to hydro-distillation for 2 hours using a Clevenger-type apparatus. The obtained essential oil was dried over anhydrous sodium sulphate, filtered and stored at +4 °C until analysis.

**Oil extraction and fatty acid methylation preparation**

Dried ground plant materials (different parts of *L. persica*) were extracted with hexane using a Soxhlet apparatus (70 °C, 8 hours) to obtain the fatty components. After removing hexane using rotary evaporator, the oily mixtures were derivatized to produce their methyl esters by trans-esterification process with 2 M methanolic KOH at 70°C for 15 minutes (Tavakoli et al., 2012; Paquat, 1992). The organic phases were analyzed by GC-FID and GC-MS systems.

**Analysis of the essential oil** **and fatty acids**

*GC-FID analysis*

The GC analysis of the essential oil and fatty acids was performed using an Agilent Technology 7890A Network gas chromatographic (GC) system, equipped with an FID detector. Compounds were separated on a DB- 5 Fused-silica capillary column (60 m long, 250 μm i.d. with 0.25μm film thickness, Agilent Technology). A sample of 1.0 μL was injected in the split mode with a split ratio of 1:5. The oven temperature was programmed to rise from 50 to 240°C at a rate of 4°C/min. 

*GC-MS analysis*

The GC-MS analysis was performed with an Agilent Technology 5975C mass-selective detector coupled to anAgilent Technology 7890A gas chromatographic. For GC–MS detection, an electron ionization system, with ionization energy of 70 eV, was used. Column oven temperature program was the same as in GC analysis. Helium was used as the carrier gas at a flow rate of 1.0 ml/min. Mass range was 30 - 600 *m/z*, while injector and MS transfer line temperatures were set at 220 °C and 250 °C, respectively. 

*Compounds identification*

The oil components were identified by calculation of their retention indices under temperature-programmed conditions for *n*-alkanes (C_6_- C_23_) and the oil on DB-5 column under the same conditions. Identification of individual compounds was done by comparison of their mass spectra with those of the internal reference mass spectra library (Wiley 7.0 n,NIST 08) or with authentic compounds and confirmed by comparing their retention indices with authentic compounds or with those reported in the literature (Davies, 1990; Shibamoto, 1987; Adams,2007).

**Antimicrobial activity**

*Microbial strains*

The essential oils were tested against two Gram-positive bacteria:* Staphylococcus aureus* ATCC 25923, and *Streptococcus* *sobrinus* ATCC 27609 and two Gram-negative bacteria including *Escherichia coli *ATCC 25922*,*and * Pseudomonas aeruginosa* ATCC 27853.

*Micro dilution broth method*

Micro-dilution susceptibility assay was performed using the NCCLS method for the determination of minimum inhibitory concentration (MIC) and minimum bactericidal concentration (MBC) (Wayne, 1999). Dilutions were prepared in 96-well microtiter plates to get final concentrations ranging from 0 to 4,000 µg/ml. All tests were performed in BHI broth medium. Bacterial cell numbers were adjusted to approximately 1 × 10^8^ CFU (colony forming units)/ml. The 96-well plates were prepared by dispensing 95 µl of nutrient broth and 5 µl of the inoculums into each well. The final volume in each well was 200 µl. The plates were incubated at 37 °C for 24 hours. Gentamicin was used as positive standard in order to control the sensitivity of the microorganisms. The growth was indicated by the presence of a white ‘pellet’ on the well bottom. The MIC was calculated as the highest dilution showing complete inhibition of the tested strains.

## Results

**Chemical Composition of the Essential Oil**

The yields of essential oils of leaf, flower, stem and root of *L. persica* were 0.32%, 1.48%, 0.65%, 0.61% (w/w % based on dry matter weight ), respectively . The essential oils of different parts of *L. persica* were obtained by hydro-distillation method and examined by GC-FID and GC–MS. The colors of essential oils were yellow to green. The results obtained from GC-FID and GC–MS analysis of the essential oils of *L. persica* were shown in Table 1. GC and GC∕MS analysis of the oils were resulted in detection 94%, 96%, 93%, 99% of the total essential oil of flowers, stems, roots and leaves, respectively. The main components of flower oil were cis-ocimene (15.4%), β-myrcene (4.4%), β-ocimene (3.9%), and γ-terpinene (5.0%). The major constituents of stem oil were β-phellandrene (5.4%), β-cymene (6.9%), and valencene (3.9%). The main compounds of roots oil were fukinanolid (17.0%), α-phellandrene (11.5%), and β-selinene (5.0%) and in the case of leaves oil were cis-ocimene (4.8%), β-ocimene (4.9%), linolenic acid, and methyl ester (4.7%).

**Fatty acid Composition**

The analysis of fatty acid obtained from different parts of *L. persica* revealed the presence of over 19 compounds as shown in Table 2. The major components were linoleic acid (10.9-31.6%), linolenic acid (4.7-21.8%) and palmitic acid (7.2-23.2%). The results demonstrated that the quantities of unsaturated fatty acids (20.4-54.7%) were higher than saturated fatty acids (9.1-28.9%). 

**Table 1:** Essential Oil composition of the different parts of *L. persica*

**Chemical Componds**	**RI** a	**%Leaf**	**%Flower**	**%Stem**	**Root % **
**α –Thujene**	927	0.2	trace	0.4	———
**α –Pinene**	939	1.3	0.6	2.6	1.8
**α –Fenchene**	952	0.1	0.1	0.4	———
**Camphene**	954	0.2	0.1	0.6	0.1
**Verbenene**	959	trace	———	trace	0.1
**β –Phellandrene**	981	2.6	———	5.4	———
**Sabinene**	982	2.7	trace	———	———
**β – Pinene**	984	0.9	0.7	2.5	3.1
**β –Myrcene**	995	2.0	4.4	2.8	0.5
**α –Phellandrene**	1007	0.6	0.2	2.7	11.5
**δ** ** - 3 Carene**	1014	trace	———	0.3	2.4
**α-Terpinene**	1019	0.3	0.1	0.6	0.2
**β –Cymene**	1026	0.8	0.2	6.9	2.3
**D-Limonene**	1030	0.9	0.2	1.6	0.9
**cis-Ocimene**	1036	4.8	15.4	1.0	———
**β –Ocimene**	1041	4.9	3.9	3.7	1.0
**γ** ** –Terpinene**	1053	0.4	5.0	0.8	2.0
**trans-Sabinene hydrate**	1057	———	———	0.2	———
**cis-Sabinene Hydrate**	1058	0.09	trace	———	———
**E-Citral**	1061	0.3	trace	0.5	———
**Linalool**	1098	0.4	trace	0.3	0.2
**Chemical Componds**	**RI** a	**%Leaf**	**%Flower**	**%Stem**	**Root % **
**Alloocimene**	1132	2.7	4.1	1.5	———
**cis-β-Terpineol**	1144	0.1	———	trace	———
**4-Terpineol**	1178	0.4	0.1	0.4	———
**α-Terpineol**	1189	0.3	trace	0.1	0.1
**Myrtenal**	1207	0.4	———	0.4	———
**Carvacrol**	1299	0.2	———	0.5	0.4
**4-Decenoic acid, methyl ester**	1311	1.8	0.2	0.7	———
**Myrtenol**	1335	0.3	———	trace	———
**(+)-4-Carene**	1357	0.4	———	0.5	———
**α –Selinene**	1363	———	———	0.1	———
**trans-Carveol**	1375	0.2	———	0.2	———
**Geranyl acetate**	1383	0.6	———	1.1	———
**β –Damascenone**	1395	0.2	———	———	———
**β –Bourbonene**	1403	———	trace	———	———
**β – Elemene**	1405	———	1.1	0.4	———
**Mentha-1,4,8-triene**	1407	0.8	———	———	———
**trans-Caryophyllene**	1426	0.3	0.3	0.8	2.0
**α-Cedrene**	1437	0.1	trace	0.1	———
**α –Amorphene**	1446	———	trace	———	———
**Germacrene B**	1447	0.1	———	———	0.3
**α –Gurjunene**	1454	———	———	trace	0.6
**β –Farnesene**	1455	0.2	1.2	0.1	———
**Thujopsene**	1456	0.7	———	———	0.7
**5,9-Undecadien-2-one**	1459	0.2	———	———	———
**β –Selinene**	1463	0.3	0.4	0.4	5.0
**Geranyl propionate**	1477	———	———	0.4	———
**β –Guaiene**	1478	0.4	———	———	0.3
**1s,Cis-Calamenene**	1486	———	———	0.2	0.2
**β –Ionone**	1430	———	———	0.1	———
**(-)-Aristolene**	1490	0.2	0.2	0.2	1.3
**γ** ** –Curcumene**	1492	———	———	1.2	———
**Germacrene-D**	1505	0.7	1.1	———	———
**Valencene**	1509	1.9	3.3	4.0	7.1
**Vitispirane**	1522	———	0.4	———	———
**γ** **–** **Cadinene**	1534	———	0.2	———	———
**β –Agarofuran**	1537	1.2	1.5	2.3	1.0
**δ** ** –Cadinene**	1540	0.3	0.1	0.1	1.5
**(E,Z)- α-Farnesene**	1543	———	0.2	———	———
**γ** ** –Gurjunene**	1544	———	———	0.1	0.4
**Cis-Α-Bisabolene**	1545	———	trace	———	1.5
**α –Agarofuran**	1550	1.2	———	2.8	———
**β –Vatirenene**	1583	0.2	0.1	———	———
**γ** ** –Elemene**	1598	0.4	———	———	———
**(+) Spathulenol**	1600	———	———	0.1	———
**(-)-Spathulenol**	1602	1.4	0.7	———	———
**Caryophyllene oxide**	1608	0.8	0.3	0.5	0.1
**Diepi-.α.-cedrene epoxide**	1609	———	———	———	1.1
**Chemical Componds**	**RI** a	**%Leaf**	**%Flower**	**%Stem**	**Root % **
**Guaiol**	1610	———	———	0.2	———
**(-)-Lepidozene**	1628	———	———	0.2	———
**γ** ** –Eudesmol**	1633	1.1	2.6	3.5	———
**β –Eudesmol**	1652	———	———	1.4	———
**β –Cadinene**	1654	3.3	3.0	0.1	0.6
**α –Eudesmol**	1656	0.8	———	———	———
**(+)-Calarene**	1658	2.7	1.7	0.2	2.1
**Hinesol**	1660	———	———	0.2	———
**Cubenol**	1663	———	trace	———	———
**Elemol**	1668	———	———	1.2	———
**Veridiflorol**	1674	3.0	———	0.3	———
**Aromadendrene**	1681	2.8	———	———	———
**β –Neoclovene**	1689	0.4	———	3.1	———
**Liguhodgsonal**	1714	1.2	———	———	0.6
**Cyercene I**	1720	3.3	2.2	2.1	1.2
**α-Ionene**	1745	———	0.1	———	———
**β –Thujone**	1775	———	———	———	2.9
**α –Guaiene**	1808	0.1	0.1	———	0.3
**Fukinanolid**	1836	———	1.0	1.3	17.0
**3,5-Dihydroxytoluene**	1856	———	3.7	1.7	———
**2,5-Furandicarboxaldehyde**	1870	2.6	———	———	0.7
**Pentadecanoic acid, ethyl ester**	1897	0.2	1.2	———	———
**Hexadecanoic acid, methyl ester**	1930	1.2	1.2	0.5	———
**Isophytol**	1951	0.1	———	0.1	———
**n-Hexadecanoic acid**	1969	0.9	0.5	1.8	0.7
**Hexadecanoic acid, ethyl ester**	1997	0.7	0.3	0.2	trace
**Linolenic acid**	2105	0.4	2.2	0.4	———
**Linolenic acid, methyl ester**	2106	4.7	0.6	1.5	———
**Phytol**	2115	trace	trace	0.1	———
**Octadecanoic acid, methyl ester**	2126	0.1	trace	trace	———
**9-Eicosyne**	2132	———	———	0.1	———
**1,19-Eicosadiene**	2138	———	———	0.1	———
**cis-9-Hexadecenal**	2155	0.1	———	———	———
**Linoleic acid ethyl ester**	2163	1.6	0.5	0.2	———
**2-Ethylhexyl trans-4-methoxycinnamate**	2169	———	———	0.2	———
**Octadecanoic acid, ethyl ester**	2194	0.1	———	———	———
**n-Docosane**	2200	trace	trace	trace	———
**1-Chloro-Nonadecane, **	2201	trace	———	———	———
**Bicyclo[10.8.0]eicosane**	2224	trace	trace	———	———
**Tricosane**	2301	0.3	0.8	0.1	———
**Monoterpene hydrocarbons** **Oxygenated monoterpenes**		28.6 4.9	35.1 0.4	34.5 4.4	25.9 3.6
**Sesquiterpen hydrocarbons** **Oxygenated sesquiterpens** **Diterpenoids**		22 7.2 0.4	17.3 4.6 0.9	18.4 9.1 0.2	26.1 18.8 ———
**Chemical Componds**	**RI** a	**%Leaf**	**%Flower**	**%Stem**	**Root % **
**Fatty acids**		12.3	7.0	4.8	0.75
**Aldehydes**		2.7	———	———	0.7
**Other hydrocarbons**		0.6	3.8	1.9	———
**Others**		20.0	25.0	22.1	17.3
**Total**		98.7	94.1	95.4	93.1

a RI: Retention index relative to n-alkanes (C_6 –_C_23_) on a DB-5 column.

**Table 2:** Fatty-acid composition of the different parts of *L. persica*

**Methyl esters**	**RT(min)**	**%** **Leaf**	**%Flower**	**%Stem**	**%Root**
**Butanoic acid, 3-methyl-, methyl ester**	9.2	0.3	0.2	———	———
**2-Butenoic acid, 2-methyl-, methyl ester**	11.01	3.6	0.8	———	2.1
**Octanoic acid, methyl ester**	23.09	———	5.9	———	———
**Dodecanoic acid, methyl ester**	36.8	———	6.1	———	0.1
**Tetradecanoic acid, methyl ester**	42.6	1.7	4.7	0.6	———
**Methyl 9-methyltetradecanoate**	45.3	———	0.2	———	———
**Pentadecanoic acid, methyl ester**	45.4	———	———	———	0.6
**Pentadecanoic acid, 14-methyl-, methyl ester**	46.9	———	———	———	0.3
**9-Hexadecenoic acid, methyl ester**	47.3	———	———	———	0.6
**Hexadecanoic acid, methyl ester**	47.9	9.5	11.7	23.2	7.2
**Hexadecanoic acid, 14-methyl-, methyl ester**	50.3	———	———	———	0.1
**Heptadecanoic acid, methyl ester**	49.4	0.7	———	1.6	0.71
**9-Octadecenoic acid, methyl ester**	51.2	———	———	———	0.3
**9,12-Octadecadienoic acid, methyl ester**	51.9	11.3	10.9	31.6	12.4
**9,12,15-Octadecatrienoic acid, methyl ester**	52.1	21.8	9.1	21.2	4.7
**Octadecanoic acid, methyl ester**	52.6	7.7	7.2	2.0	0.3
**Eicosanoic acid, methyl ester**	58.5	1.0	———	1.1	———
**Docosanoic acid, methyl ester**	67.6	———	0.1	———	———
**other hydrocarbon compounds identified**		42.3	43.2	18.8	70.5
**∑Saturated fatty acids** **∑Unsaturated fatty acids**		13.2 44.4	28.9 27.1	26.4 54.7	9.1 20.4

**Inhibition of Bacterial Growth**

The anti-bacterial activity of the essential oil from *L. persica* against a panel of pathogenic microorganisms was assessed by measurement of minimum inhibitory concentration (MIC). The results are presented in Table 3. It can be concluded that the essential oil of root has the highest anti-bacterial activity and the oil of the leaves has the least efficient antibacterial activity among other parts. The Gram-negative bacterium that exhibited a higher sensitivity to the tested oils was *Pseudomonas aeruginosa*. The essential oil from stems showed the highest anti-bacterial effect against *Pseudomonas aeruginosa* (7.16 *μ*g/ml in terms of MIC) and the least anti-bacterial activity was seen for leaves essential oil against *Staphylococcus aureus *(375 *μ*g/ml in terms of MIC). 

**Table3.** MIC (µg/ml) of the *Ligulari apersica *essential oils.

**Types of bacteria**	**Gram negative**		**Gram positive**	
**Microorganism:** **ATCC number:**	*Pseudomonas aeruginosa* 27853	*Escherichia coli * 25922	*Streptococcus sobrinus* 27609	*Staphylococcus aureus* 25923
**Flowers essential oil**	7.8	250	15.6	62.5
**Leaves essential oil**	23.4	187.5	187.5	375
**Stems essential oil**	7.2	114.7	114.7	28.7
**Roots essential oil**	15.6	62.5	31.2	31.2
**gentamicin**	1	1	0.2	0.5

MIC= Minimum Inhibitory Concentration

## Discussion

A comparison between reported chemical composition of the aerial parts of *L. persica *showed that the similar composition were obtained (Mirjalili and Yousefzadi, 2012). In general, monoterpenes and sesquiterpenes were more abundant as compared to the other compounds. In addition, the presence of significant amounts of various bioactive constituents indicates a possible industrial use of these plants. Fukinanolid or bakkenolide A (17.0%), as the most abundant sesquiterpene in roots, α-pinen have been recently introduced as a powerful anti-microbial and anti-tumor agent (Rustaiyan et al., 1999). Cis-ocimen that was the most abundant chemical in flowers (15.4%) is used as raw material in perfumes and cosmetics. Therefore, the essential oils of *L. persica* are suitable as natural supplement sources for food, cosmetic and pharmaceutical industries.

In addition, the amounts of the unsaturated fatty acids in the leaves and stems were higher than of the flowers and roots. Unsaturated fatty acids play a crucial role in human nutrition and health. Polyunsaturated fatty acids (PUFAs) have been considered as health-promoting nutrients in recent years. A growing body of studies illustrates the benefits of PUFAs in alleviating cardiovascular, inflammatory, heart diseases, atherosclerosis, autoimmune disorder, diabetes and other diseases (Finley, 2001).

Our study reported the secondary metabolites in essential oil and fatty acids extracted from different parts of *Ligularia persica*, as well as their antibacterial activities. These results indicate that *L. persica* may be a rich source of natural products with biological activities.
